# Effects of activation energy and chemical reaction on unsteady MHD dissipative Darcy–Forchheimer squeezed flow of Casson fluid over horizontal channel

**DOI:** 10.1038/s41598-023-29702-w

**Published:** 2023-02-15

**Authors:** Shuguang Li, Kodi Raghunath, Ayman Alfaleh, Farhan Ali, A. Zaib, M. Ijaz Khan, Sayed M. ElDin, V. Puneeth

**Affiliations:** 1 School of Computer Science and Technology, Shandong Technology and Business University, Yantai, 264005 China; 2 St John’s College of Engineering and Technology, Yemmiganur, Kurnool District, Andhra Pradesh 518360 India; 3 College of Engineering, Industrial Engineering Department, Umm Al-Qura University, 28821 Al-Khalidiya District, Al-Qunfudhah City, Kingdom of Saudi Arabia; 4 Department of Mathematical Sciences, Federal Urdu University of Arts, Sciences & Technology, Gulshan-e-Iqbal Karachi, 7530 Pakistan; 5 Department of Mechanical Engineering, Lebanese American University, Beirut, Lebanon; 6 Department of Mathematics and Statistics, Riphah International University I-14, Islamabad, 44000 Pakistan; 7 Center of Research, Faculty of Engineering, Future University in Egypt, New Cairo, 11835 Egypt; 8 Department of Computational Science, CHRIST (Deemed to Be University), Ghaziabad, 201003 India

**Keywords:** Engineering, Mathematics and computing

## Abstract

The impact of chemical reaction and activation energy plays a vital role in the analysis of fluid dynamics and its thermal properties. The application of the flow of fluid is significantly considered in nuclear reactors, automobiles, manufacturing setups, electronic appliances etc. This study explores the impacts of activation energy and chemical reaction on the magnetohydrodynamic Darcy–Forchheimer squeezed Casson fluid flow through a porous material across the horizontal channel where the two parallel plates are assumed to be in motion. By using similarity variables, partial differential equations are converted to ordinary differential equations. Numerical method is applied using MATLAB to solve the problems and acquire the data for velocity field, thermal distribution, and concentration distribution. The graphs indicate that fluid velocity and temperature increases as the plates are brought closer. In addition, there was a correlation between a rise in the Hartmann number and a decrease in the fluid's velocity because of the existence of strong Lorentz forces. The temperature and the concentration of the liquid will increase due to the Brownian motion. When the Darcy–Forchheimer and activation energy parameters are both increased, the velocity and concentration decreases.

## Introduction

Squeezing flow between two parallel discs has garnered much interest recently due to the wide range of applications in technical and industrial setups. The notion of flow between two squeezing surfaces is used in devices like, hydraulic brakes, the moving piston of an engine, chocolate fillers, and many more. Syringes and nasogastric tubes both include the process of squeezing flow while a moving disc is influencing it. A more profound understanding of these fluxes results in creating more effective and efficient machines that may be used for various mechanical and industrial applications. The manufacturing of hydrodynamical devices, accelerators, compression and injection moulding, lubrication equipment, and polymer processing are some of the places where squeezing flow can be observed. Stefan^1^ studied squeezing flow using the lubrication approximation; several scholars subsequently examined squeezing flow issues for various geometrical configurations using multiple approaches. Moore^2^ indicated that the influences such as surface finish, viscoelastic liquids, elastomeric surfaces, and molecular effects plays a vital role and hence it must be considered either partially or entirely depending on the degree of complexity of the problems. Gupta et al.^3^ noticed that the unsteady squeezing channel flow problem could be significantly simplified via similarity variables. The distance between the paralleled plates varies as the square root of a linear function of time. In this scenario, the similarity variables allow the problem to be significantly simplified. Duwairi et al.^4^ studied the effects of heat transfer on unstable squeezing channel flow, they assumed that the paralleled walls were heated evenly at a constant temperature. This allowed them to examine the impact of heat transfer on the flow. Furthermore, various scholars have looked into the heat transfer properties of nanofluid flowing between parallel plates^5–7^ by considering various physical conditions.

Squeezing flow between parallel plates finds its significance in the area of fluid dynamics as it finds applications in hydraulic machinery and tools, electric motors, food industry, bioengineering, and automobile engines. Other simpler but equally important examples are flow patterns occurring in syringes and compressible tubes. In these applications, flow patterns can be classified into laminar, turbulent, and transitional flows on the basis of the well-known Reynold’s number. From an industrial perspective, it is necessary to study the effect of these different behaviors for non-Newtonian fluids and in this regards many scholars have studied the flow of Casson fluid^8,9^ as it is able to capture complex rheological properties of a fluid. It was observed that the movement of microorganisms within the Casson nanofluid will help in preventing the agglomeration of nanoparticles and provides a smoother flow^10,11^. Concentrated fluids like sauces, honey, juices, blood, and printing inks can be well described using this model. Casson fluid can be defined as a shear thinning liquid which is assumed to have an infinite viscosity at zero rate of shear, a yield stress below which no flow occurs, and a zero viscosity at an infinite rate of shear. Hussain et al.^12^ performed a non-similar analysis to study the EMHD flow of Casson nanofluid considering the shape of the suspended nanoparticle as a factor. Jamshed et al.^13^ implemented the Tiwari-Das model to examine the thermal properties of Casson nanofluid and found an increase in the temperature absorbed when the nanoparticle volume fraction was raised. Furthermore these studies were extended to anaylse the motion of Casson nanofluid over a Riga plate by Upreti et al.^14^.

The widespread use of mass transport with activation energy in essential fields such as geothermal engineering, chemical engineering, oil emulsions, and food processing has drawn the attention of researchers. Arrhenius proposed the concept of activation energy in the year 1889. It is the minimal amount of energy that particles must obtain to undergo a chemical reaction. This energy might exist in kinetic or potential energy, and without it, reactants cannot make products. Activation energy has a comprehensive range of applications, including geothermal engineering, chemical engineering, oil emulsions, and food processing. In the first part of his study, Bestman^15^ looked at the convective flow of binary amalgam through a porous medium. Makinde et al.^16^ investigated the activation energy and the impacts of the nth order chemical process on a time-dependent radiated flat porous panel. Alsaadi et al.^17^ studied the nonlinear mixed convective flow of non-Newtonian nanoliquids across an absorbent stretched sheet. In contrast, the flow was subjected to the influence of nonlinear radiation and activation energy. Additionally, the researchers explored the rate at which entropy is generated. The researchers in this study concluded that an increase in the activation energy parameter led to a rise in concentration. Irfan et al.^18^ constructed an unsteady flow of Carreau nanofluid to gain the impacts of binary chemical reaction and activation energy. They reported the alterations in shear-thinning fluids and shear thickening fluids with the influence of the reaction rate parameter, revealing that the concentration decreased.

The amount of energy that must be present in a chemical system containing potential reactants to produce a chemical reaction is referred to as the activation energy. The Arrhenius equation, which explains the shift in rate constants as a function of temperature, is the formula used to calculate activation energy. In geothermal engineering, chemical engineering, mechano-chemistry, oil and water emulsions, and the degradation of materials, a mass transfer phenomenon with a chemical reaction is employed. Chemical reactions and mass transfer have a complicated relationship with one another. This connection may be investigated for both fluid flow and mass transfer by fabricating and digesting reactant species at varying speeds, which both involve the flow of fluids and the transfer of mass. Hsiao^19^ gave a numerical analysis of the manufacturing efficiency of a thermal extrusion system by applying an improved method of controlling parameters. This was accomplished via the use of an enhanced technique of controlling parameters. Majeed et al.^20^ investigated the accumulative impacts of a binary chemical reaction and activation energy in a fluid flow under a scenario of second-order momentum slip. Along with the topic of activation energy, Khan et al.^21^ examined the influence of nonlinear thermal radiation. They found a correlation between the more significant activation energy parameter and a rise in species concentration. Dhlamini et al.^22^ broadened the scope of the study by taking into account mixed convection. They discovered that using a heated plate increased the concentration of the chemical species. For the flow of Carreau fluids, Irfan et al.^18^ used nonlinear mixed convection as their transport mechanism. The nonlinear fluctuation of density, as opposed to the linear variation of density, may sometimes lead to a more significant increase in species concentration.

Concerning the petroleum industry, convective fluid flow phenomena in a porous space are essential when considering high flow rate regions, likely to occur near wellbores for gas and condensate reservoirs. This is because these regions will likely be where high flow rates arise. High velocities may be found in several contemporary applications that use porous areas. A modified version of the traditional Darcy's law, known as the non-Darcian porous space, considers the effects of both porous space and inertia. The majority of the research that has been done on this topic has modelled and analysed flow issues across porous areas using the traditional Darcy's equation. However, Darcy's theory in its classical form breaks down under the conditions of greater velocities and bigger porosities. Therefore, to take into account inertia's effects, Forchheimer^23^ included a square velocity factor in the equation for momentum. This component was referred to as "Forchheimer's word" in Muskat's^24^ analysis. Seddeek's^25^ used the Darcy–Forchheimer relation to investigate the mixed convective flow of nanofluid. Jha and Kaurangini^26^ discovered approximate solutions for the nonlinear Brinkman– Forchheimer-extended Darcy flow model they were working on. Pal and Mondal^27^ investigated the hydromagnetic Darcy–Forchheimer flow of a liquid with varying viscosity. Darcy–Sadiq and Hayat^28^ examined the Forchheimer flow of a magneto-Maxwell liquid limited by a convectively heated sheet. Shehzad et al.^29^ investigated the impact of the Cattaneo–Christov heat flux model on the Darcy–Forchheimer flow of an Oldroyd–B fluid with variable conductivity and nonlinear convection. Bakar et al.^30^ investigated the forced convection boundary layer stagnation-point flow in a Darcy–Forchheimer porous space towards a diminishing sheet. Hayat et al.^31^ studied a Maxwell material's Darcy–Forchheimer flow when it was subjected to a heat flux. Their research was based on the Cattaneo–Christov theory and included variable thermal conductivity. Using the Darcy–Forchheimer–Brinkman model, Umavathi et al.^32^ performed a computational analysis of the natural convective flow of nanofluids and the heat transfer that occurred inside a vertical rectangular duct. The Darcy–Forchheimer flows of viscoelastic nanofluids were the subject of comparative research carried out by Hayat et al.^33^. A recently improved model for the Darcy–Forchheimer flow of a Maxwell nanofluid with a convective surface condition was published by Muhammad et al.^34^.

In this paper, we analyse the effects of activation energy and chemical reaction on magnetohydrodynamic (MHD) Darcy–Forchheimer squeezing Casson fluid flow across porous media along a horizontal channel. Using similarity variables, one may successfully convert partial differential equations (PDEs) to ordinary differential equations (ODEs). Numerical method is applied using MATLAB to solve the problems and acquire the data for velocity field, thermal distribution, and concentration distribution. Results have been plotted graphically. In addition, the correctness of the solution has been checked by comparing them to the results published in peer-reviewed papers.

## Formulation of the problem

**Figure 1:**
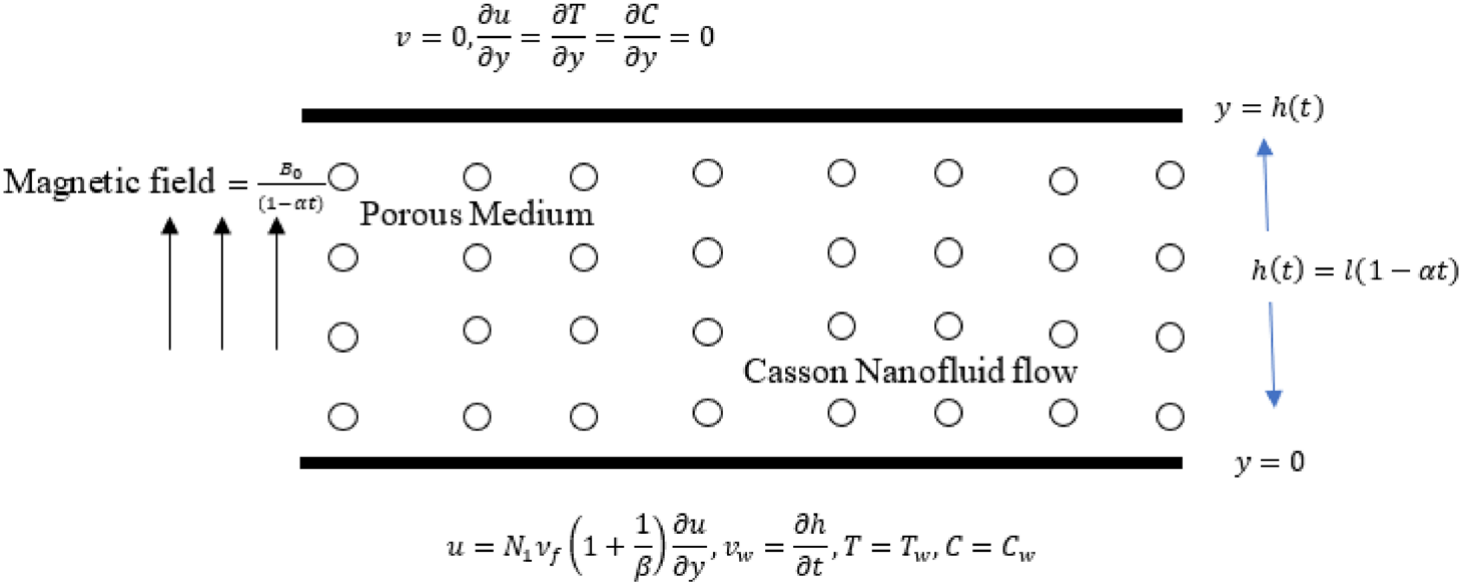
Physical model of problem.

The symmetric and time-dependent Magnetohydrodynamic Darcy–Forchheimer squeezed Casson nanofluid flow through a porous material with activation energy and chemical reaction is presented. The movement of water through the channel is caused by the compression of two of its surfaces. The separation between the upper and lower surface if *y* = ℎ(*t*) = (1 − α*t*)^1/2^. The magnetic field B(t) is assumed to be perpendicular to the bottom plate^35^. The geometrical representation of the Casson fluid flow is shown in Fig. 1. In addition, the influence of a homogeneous chemical reaction of the first order is considered in the concentration equation. Given these assumptions, the continuity, momentum, energy, and concentration equations that regulate the current physical issue with required circumstances are as follows^36^:

1 $$\frac{\partial u}{{\partial x}} + \frac{\partial u}{{\partial y}} = 0$$

2 $$\frac{\partial u}{{\partial t}} + u\frac{\partial u}{{\partial x}} + v\,\frac{\partial u}{{\partial y}} = - \frac{1}{{\rho_{f} }}\frac{\partial P}{{\partial x}} + v_{f} \left( {1 + \frac{1}{\beta }} \right)\left( {\frac{{\partial^{2} u}}{{\partial x^{2} }} + \frac{{\partial^{2} u}}{{\partial y^{2} }}} \right) - \frac{{\sigma B^{2} (t)}}{{\rho_{f} }}u - v_{f} \left( {1 + \frac{1}{\beta }} \right)\frac{\varphi \,}{{k_{1} (t)}}u - \frac{{C_{p} }}{{x\sqrt {K^{ * } } }}u^{2}$$

3 $$\frac{\partial v}{{\partial t}} + u\frac{\partial v}{{\partial x}} + v\,\frac{\partial v}{{\partial y}} = - \frac{1}{{\rho_{f} }}\frac{\partial P}{{\partial y}} + v_{f} \left( {1 + \frac{1}{\beta }} \right)\left( {\frac{{\partial^{2} v}}{{\partial x^{2} }} + \frac{{\partial^{2} v}}{{\partial y^{2} }}} \right)$$

4 $$\begin{gathered} \frac{\partial T}{{\partial t}} + u\frac{\partial T}{{\partial x}} + v\,\frac{\partial T}{{\partial y}} = \alpha_{f} \left( {\frac{{\partial^{2} T}}{{\partial x^{2} }} + \frac{{\partial^{2} T}}{{\partial y^{2} }}} \right) + \tau \left[ {\frac{{D_{B} }}{{C_{\infty } }}\left( {\frac{\partial C}{{\partial y}}\frac{\partial T}{{\partial y}} + \frac{\partial C}{{\partial x}}\frac{\partial T}{{\partial x}}} \right) + \frac{{D_{T} }}{{T_{\infty } }}\left( {\frac{\partial T}{{\partial x}} + \frac{\partial T}{{\partial y}}} \right)^{2} } \right] \hfill \\ + \frac{{v_{f} }}{{c_{f} }}\left( {1 + \frac{1}{\beta }} \right)\left[ {4\left( {\frac{\partial u}{{\partial x}}} \right)^{2} + \left( {\frac{\partial u}{{\partial y}}} \right)^{2} } \right] + \frac{{\sigma B_{0}^{2} (t)}}{{\left( {\rho c} \right)_{f} }}u^{2} - \frac{1}{{\left( {\rho c} \right)_{f} }}\left( {\frac{{\partial q_{r} }}{\partial x} + \frac{{\partial q_{r} }}{\partial y}} \right) + \frac{{Q_{0} }}{{\left( {\rho c} \right)_{f} }}\left( {T - T_{\infty } } \right) \hfill \\ \end{gathered}$$

5 $$\frac{\partial C}{{\partial t}} + u\frac{\partial C}{{\partial x}} + v\,\frac{\partial C}{{\partial y}} = D_{B} \left( {\frac{{\partial^{2} C}}{{\partial x^{2} }} + \frac{{\partial^{2} C}}{{\partial y^{2} }}} \right) + \frac{{D_{T} C_{\infty } }}{{T_{\infty } }}\left( {\frac{{\partial^{2} T}}{{\partial x^{2} }} + \frac{{\partial^{2} T}}{{\partial y^{2} }}} \right) - k_{r}^{2} \left( {C - C_{\infty } } \right)\left( {\frac{T}{{T_{\infty } }}} \right)^{m} \exp \left( {\frac{{ - E_{a} }}{{K_{1} T}}} \right),$$

The correlated boundary conditions (BCs) are

6 $$u = N_{1} v_{f} \left( {1 + \frac{1}{\beta }} \right)\frac{\partial u}{{\partial y}},\,v = v_{w} = \frac{\partial h(t)}{{\partial t}},\,T = T_{w} ,C = C_{w} ,\,\,y = 0$$

7 $$\frac{\partial u}{{\partial y}} = 0,\,\,\,\,\,v = 0,\,\,\,\,\frac{\partial T}{{\partial y}} = 0,\,\,\,\,\,\,\frac{\partial C}{{\partial y}} = 0,\,\,\,y = h\left( t \right)\,\,\,\,\,$$

The Rosseland approximation can be used for the radiative heat flux vector ${q}_{r}$ because there is also self-absorption in addition to emission for an optically thick fluid. Since the absorption coefficient is typically wavelength dependent and significant, we can use the Rosseland approximation. Therefore, the definition of ${q}_{r}$ is^37,49^.

8 $$q_{r} = \frac{{ - 4\sigma_{1} }}{{3k_{1} }}\frac{{\partial T^{4} }}{\partial y}$$

In this equation, k_1_ denotes the Rosseland mean absorption co-efficient and σ_1_ stands for the Stefan–Boltzmann constant.

We are working under the assumption that the temperature changes inside the flow are not very significant, allowing us to describe T^4^ as a linear function. We extend T^4^ about the free stream temperature T using Taylor's series, ignoring higher order variables in the process. The following is an approximation that may be derived from this:

9 $$T^{4} \approx 4T_{\infty }^{3} T - 3T_{\infty }^{4}$$

The equation for energy may be obtained by combining Eqs. (8) and (9), as shown in the following:

10 $$\begin{gathered} \frac{\partial T}{{\partial t}} + u\frac{\partial T}{{\partial x}}v\,\frac{\partial T}{{\partial y}} = \alpha_{f} \left( {\frac{{\partial^{2} T}}{{\partial x^{2} }} + \frac{{\partial^{2} T}}{{\partial y^{2} }}} \right) + \tau \left[ {\frac{{D_{B} }}{{C_{\infty } }}\left( {\frac{\partial C}{{\partial y}}\frac{\partial T}{{\partial y}} + \frac{\partial C}{{\partial x}}\frac{\partial T}{{\partial x}}} \right) + \frac{{D_{T} }}{{T_{\infty } }}\left( {\frac{\partial T}{{\partial x}} + \frac{\partial T}{{\partial y}}} \right)^{2} } \right] \hfill \\ + \frac{{v_{f} }}{{c_{f} }}\left( {1 + \frac{1}{\beta }} \right)\left[ {4\left( {\frac{\partial u}{{\partial x}}} \right)^{2} + \left( {\frac{\partial u}{{\partial y}}} \right)^{2} } \right]\,\, + \,\frac{{\sigma B_{0}^{2} (t)}}{{\left( {\rho c} \right)_{f} }}u^{2} + \alpha_{f} \left( {\frac{{16\sigma T_{\infty }^{3} }}{{3k_{f} k_{1}^{*} }}} \right)\left( {\frac{{\partial^{2} T}}{{\partial x^{2} }} + \frac{{\partial^{2} T}}{{\partial y^{2} }}} \right) + \frac{{Q_{0} }}{{\left( {\rho c} \right)_{f} }}\left( {T - T_{\infty } } \right) \hfill \\ \end{gathered}$$

The following similarity transformation is employed to obtain the ODEs from the PDEs:

11 $$\eta = \frac{y}{{l\sqrt {\left( {1 - \alpha t} \right)} }},u = \frac{\alpha x}{{2\left( {1 - \alpha t} \right)}}\,f^{\prime}(\eta ),v = \frac{ - \alpha l}{{2\sqrt {\left( {1 - \alpha t} \right)} }}\,f(\eta ),\theta = \frac{{T - T_{\infty } }}{{T_{w} - T_{\infty } }},\varphi = \frac{{C - C_{\infty } }}{{C_{w} - C_{\infty } }}$$

where η is the local similarity variable, *f*(η), θ(η) and ϕ(η) are the dimensionless velocity, temperature and concentration of the fluid in the boundary layer region, respectively.

In order to produce the following non-dimensional equations, substitute Eq. (11) into Eqs. (2), (3), and (10) respectively, and then gives.

12 $$\left( {1 + \frac{1}{\beta }} \right)\,f^{iv} - S\left( {\eta \,f^{\prime\prime\prime} + 3f^{\prime\prime} + f^{\prime}f^{\prime\prime} - ff^{\prime\prime\prime}} \right) - Ha^{2} f^{\prime\prime} - \left( {1 + \frac{1}{\beta }} \right)\frac{1\,}{{Da}}f^{\prime\prime} - F_{r} \left( {f^{\prime\prime}} \right)^{2} = 0$$

13 $$\begin{gathered} \left( {1 + \frac{4}{3}R_{d} } \right)\theta^{\prime\prime} + \Pr \,S\left( {f\theta^{\prime} - \eta \theta^{\prime} + Q\theta } \right) + \Pr \,E_{c} \left[ {\left( {1 + \frac{1}{\beta }} \right)\left[ {\left( {f^{\prime\prime}} \right)^{2} + 4\delta^{2} \left( {f^{\prime}} \right)^{2} } \right] + Ha^{2} \left( {f^{\prime}} \right)^{2} } \right] + \hfill \\ \,\,\,\,\,\,\,\,\,\,\,\,\,\,\,\,\,\,\,\,\,\,\,\,\,\,\,\,\,\,\,\,\,\,\,\,\,\,\,\,\,\,\,\,\,\,\,\,\,\,\,\,\,\,\,\,\,\,\,\,\,\,\,\,\,\,\,\,\,\,\,\,\,\,\,\,\,\,\,\,\,\,\,\,\,\,\,\,\,\,\,\,\,\,\,\,\,\,\,\,\,\,\,\,\,\,\,\,\,\,\,\,\,\,\,\,\,\,\,\,\,\,\,\,\,\,\,\,\,\,\,\,\,\,\,\,\,\,\,\,\,\,\,\,\,\,\,\left[ {\,N_{b} \phi^{\prime}\theta^{\prime} + N_{t} \left( {\theta^{\prime}} \right)^{2} } \right] = 0 \hfill \\ \end{gathered}$$

14 $$\varphi^{\prime\prime} + L_{e} S\left( {f\varphi^{\prime} - \eta \varphi^{\prime}} \right) + \frac{{N_{t} }}{{N_{b} }}\theta ^{\prime\prime} - L_{e} S_{\,} \psi \left( {1 + r_{1} \theta } \right)^{m} \exp \left( {\frac{ - E}{{1 + r_{1} \theta }}} \right)\,\varphi = 0$$

The associated dimensionless boundary conditions are as follows:

15 $$f\left( \eta \right) = 0,\,\,f^{\prime\prime}\left( \eta \right) = 0,\,\,\theta^{\prime}\left( \eta \right) = 0,\,\,\varphi^{\prime}\left( \eta \right) = 0\,\,{\text{at}}\,\,\eta \, = \,0$$

16 $$f\left( \eta \right) = 1,\,\,f^{\prime}\left( \eta \right) = \gamma \left( {1 + \frac{1}{\beta }} \right)f^{\prime\prime}\left( \eta \right),\,\,\theta \left( \eta \right) = 1,\,\,\varphi \left( \eta \right) = 0\,\,\,at\,\,\,\eta = 1$$

In the equations that do not include dimensions, the important parameters are defined as

$$\begin{aligned} & S = \frac{{\alpha l^{2} }}{{2v_{f} }},\,\,Ha = lB_{0} \sqrt {\frac{\alpha }{{\rho_{f} v_{f} }}} ,\,\,Da = \frac{{k_{0} }}{{\varphi l^{2} }},\,\,Q = \frac{{2Q_{0} }}{{\alpha \left[ {\rho C} \right]_{f} }},\,\,\delta = \frac{1}{x}\left( {1 - \alpha t} \right)^{1/2} ,\,\,\Pr = \frac{{v_{f} }}{{\alpha_{f} }} \\ & D_{a} = \frac{{k_{1} }}{{\varphi \,l^{2} }},\,\,Ec = \frac{{\alpha^{2} x^{2} }}{{4c_{f} T_{w} \left( {1 - \alpha t} \right)^{2} }},F_{r} = \frac{{C_{p} }}{\sqrt K },\,R_{d} = \frac{{4\sigma T_{\infty }^{3} }}{{k_{f} k_{1} }},L_{e} = \frac{{v_{f} }}{{D_{B} }},\,E = \frac{{E_{a} }}{{K_{1} T_{\infty } }},\,\psi = \frac{{k_{r}^{2} }}{\alpha } \\ & N_{b} = \frac{{\tau D_{B} \left( {C_{w} - C_{\infty } } \right)}}{{v_{f} C_{\infty } }},N_{t} = \frac{{\tau D_{T} (T_{w} - T_{\infty } )}}{{v_{f} T_{\infty } }},\,\gamma = \frac{{N_{1} v_{f} }}{l},r_{1} = \left( {\frac{{T_{w} - T_{\infty } }}{{T_{\infty } }}} \right) \\ \end{aligned}$$

## Physical quantities of Interests

The local skin friction coefficient Cf_x_, the local Nusselt number Nu_x_, and the local Sherwood number Sh_x_ are the physical quantities of relevance that influence the flow. These numbers have the following definitions:

17 $$\begin{gathered} Cf_{x} = \frac{1}{{\rho_{f} }}\frac{{\tau_{w} }}{{u_{w}^{2} }},\,\, \hfill \\ Nu_{x} = \frac{1}{{\alpha_{f} }}\frac{{lq_{w} }}{{\,\left( {T_{w} - T_{\infty } } \right)}},\,\, \hfill \\ Sh_{x} = \frac{1}{{D_{B} }}\frac{{lq_{s} }}{{\left( {C_{w} - C_{\infty } } \right)}} \hfill \\ \end{gathered}$$

where τ_w_, q_w_ and q_s_ are the wall skin friction, wall heat flux and wall mass flux respectively given by

18 $$\tau_{w} = \mu_{B} \left( {1 + \frac{1}{\beta }} \right)\left[ {\frac{\partial u}{{\partial y}}} \right]_{y = h(t)} ,\,\,\,\,\,\,\,\,\,\,\,q_{w} = - \alpha_{f} \left[ {\frac{\partial T}{{\partial y}}} \right]_{y = h(t)} ,\,\,\,\,\,\,\,\,q_{s} = - D_{B} \left[ {\frac{\partial C}{{\partial y}}} \right]_{y = h(t)}$$

The coefficient of skin friction, the Nusselt number, and the Sherwood number are all expressed in their non-dimensional versions in terms of the similarity variable as follows:

19 $$\left. \begin{gathered} \frac{{l^{2} }}{{x^{2} }}\left( {1 - \alpha t} \right)\sqrt {{\text{Re}}_{x} } Cf_{x} = \left( {1 + \frac{1}{\beta }} \right)\,f^{\prime\prime}(1),\, \hfill \\ \sqrt {\left( {1 - \alpha t} \right)} \,\frac{{Nu_{x} }}{{\sqrt {{\text{Re}}_{x} } }} = - \left( {1 + \frac{4}{3}R_{d} } \right)\theta^{\prime}(1),\, \hfill \\ \sqrt {\left( {1 - \alpha t} \right)} \,\frac{{Sh_{x} }}{{\sqrt {{\text{Re}}_{x} } }} = - \phi^{\prime}(1),\,\, \hfill \\ \end{gathered} \right\}$$

where *Re*_*x*_ = l*v*_*w*_/ν_*f*_ is the local Reynolds number based on the squeezing velocity *v*_*w*_.

## ND-solve (shooting) procedure

In computational analysis, the ND-Solve (Shooting) procedure is a procedure is a technique for tacking a boundary value problem (BVP) by reducing it to first order differential equation (Initial Value Problem IVP). It comprises finding solutions to the IVP for various initial conditions until one finds the solution that also fulfils the boundary conditions of the BVP. For the consider flow problem, the system of Eqs. (12–14) with boundary conditions (15, 16) are solved numerically with the help of ND-Solve (Shooting) technique. For this purpose, the higher order differential equations are first altered into first order with the help of new transformations. The new transformations procedure are listed as:

20 $$\left. {\begin{array}{*{20}c} {f = P_{1} ,\, \, f^{\prime} = P_{2} ,\, \, f^{\prime\prime} = P_{3} ,\, \, f^{\prime\prime\prime} = P_{4} ,f^{(iv)} = PP_{1} } \\ \begin{gathered} \theta = P_{5} ,\, \, \theta^{\prime} = P_{6} ,\, \, \theta^{\prime\prime} = PP_{2} , \hfill \\ \phi = P_{7} ,\, \, \theta^{\prime} = P_{8} ,\, \, \phi ^{\prime\prime} = PP_{3} , \hfill \\ \end{gathered} \\ \end{array} } \right\}\,$$

The Eqs. (12, 13, 14) with boundary conditions (15) takes the form

21 $$PP_{1} = \frac{1}{{\left( {1 + \frac{1}{\beta }} \right)}}\left[ {S\left( {\eta P_{4} + 3P_{3} + P_{2} P_{3} - P_{1} P_{4} } \right) + \left( {Ha} \right)^{2} P_{2} + \left( {1 + \frac{1}{\beta }} \right)\frac{1}{Da}P_{3} - F_{r} P_{3}^{2} } \right],$$

22 $$PP_{2} = \frac{ - 1}{{\left( {1 + \frac{4}{3}R_{d} } \right)}}\left[ {P_{r} S\left( {P_{1} P_{6} - \eta P_{6} + QP_{5} } \right) + P_{r} E_{c} \left( {\left( {1 + \frac{1}{\beta }} \right)\left( {P_{3}^{2} + 4\delta^{2} P_{2}^{2} } \right) + \left( {Ha} \right)^{2} P_{2}^{2} } \right) + N_{b} P_{6} P_{8} + N_{t} P_{6}^{2} } \right],$$

23 $$PP_{3} = - L_{e} S\left( {P_{1} P_{8} - \eta P_{8} } \right) - \frac{{N_{t} }}{{N_{b} }}PP_{2} + L_{e} S\psi \left( {1 + \gamma_{1} P_{5} } \right)^{m} P_{7} \exp \left( {\frac{ - E}{{\left( {1 + \gamma_{1} P_{5} } \right)}}} \right),$$

with

24 $$\left. {\begin{array}{*{20}c} {P_{1} (0) = 0,\, \, P_{3} (0) = 0,\, \, P_{6} (0) = 0,P_{8} (0) = 0,} \\ {P_{1} (1) = 1,\, \, P_{2} (1) - \gamma \left( {1 + \frac{1}{\beta }} \right)P_{3} (1) = 0,P_{5} (1) = 1,P_{7} (1) = 1} \\ \end{array} } \right\}\,$$

## Results and discussion

The system whose equations are Eqs. (12)–(14) has been numerically solved. In this example, a ND-Solve (Shooting) technique is utilized. An algorithm is designed in the program known as MATLAB to create both numerical and graphical answers. The findings are compared with those obtained by Noor et al.^36^ and Naduvinamani and Shankar^38^ to verify the precision of the current numerical scheme as shown in Table 1. In addition, it can be seen from Table 1 that the absolute values of wall shear stress go up as the values of the squeezing number go up, whilst the Nusselt and Sherwood numbers go down. This is the opposite of what happens when the squeezing number goes down. Additionally, it should be no surprise that the passage of heat from the surface of parallel plates to the fluid in between the plates is indicated by the Nusselt number having negative values.

**Table 1:** Comparison of − *f* ′′ (1), − θ ′ (1) and − ϕ ′ (1) for different values of *S* when β → ∞, *Da* → ∞ and *Ha* = *Rd* = γ = *Nb* = *Nt* = E = Fr = 0.

S	Noor et al.^36^			Naduvinamani and Ushashanka^38^			Present Values		
	−*f* ′′(1)	−θ′ (1)	−ϕ′ (1)	−*f* ′′(1)	−θ′ (1)	−ϕ′ (1)	−*f* ′′(1)	−θ′ (1)	−ϕ′ (1)
− 1.0	2.170255	3.319904	0.804558	2.170091	3.319899	0.804559	2.171247	3.312257	0.878521
− 0.5	2.617512	3.129556	0.781404	2.617404	3.129491	0.781402	2.615452	3.123214	0.788521
0.01	3.007208	3.047166	0.761229	3.007134	3.047092	0.761225	3.045871	3.048521	0.761214
0.5	3.336504	3.026389	0.744229	3.336449	3.026352	0.744224	3.337521	3.026521	0.744323
2.0	4.167412	3.118564	0.701819	4.167389	3.118551	0.701813	4.165512	3.116521	0.701211

**Figure 2:**
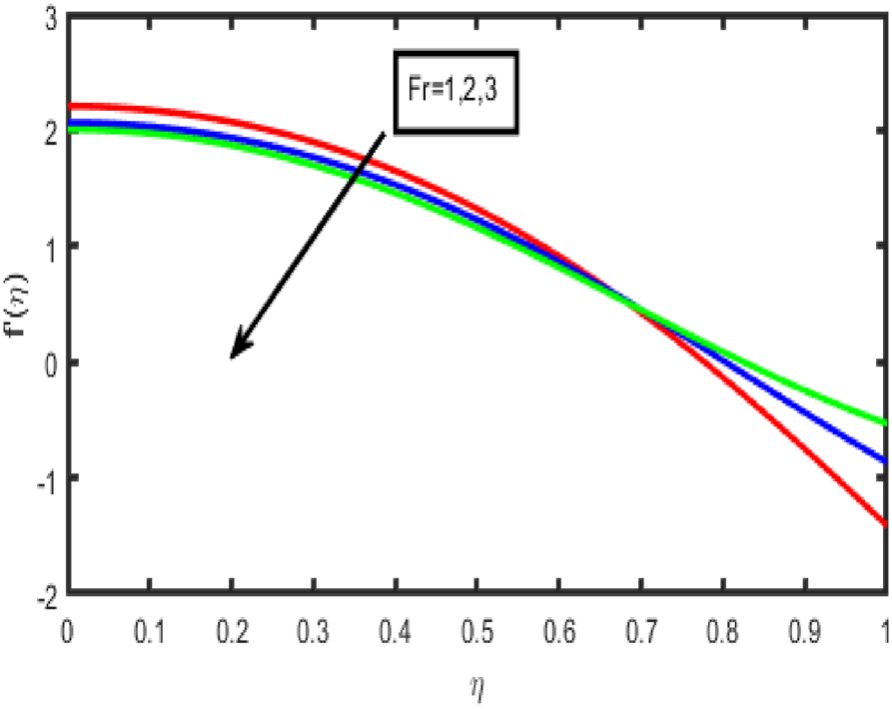
Effect of F_r_ on f(n).

**Figure 3:**
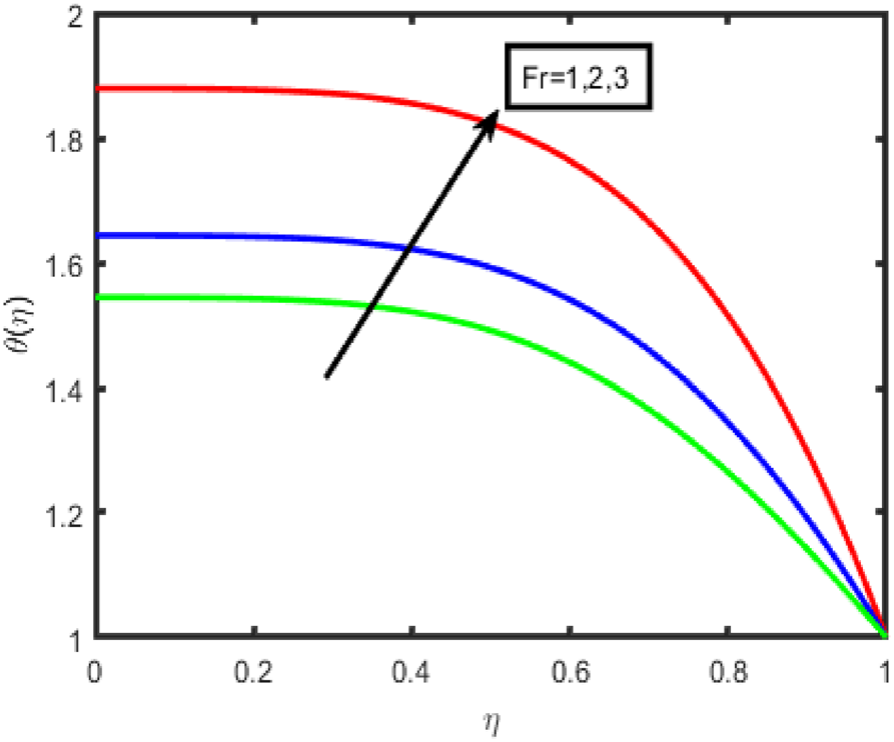
Effect of F_r_ on temperature.

**Figure 4:**
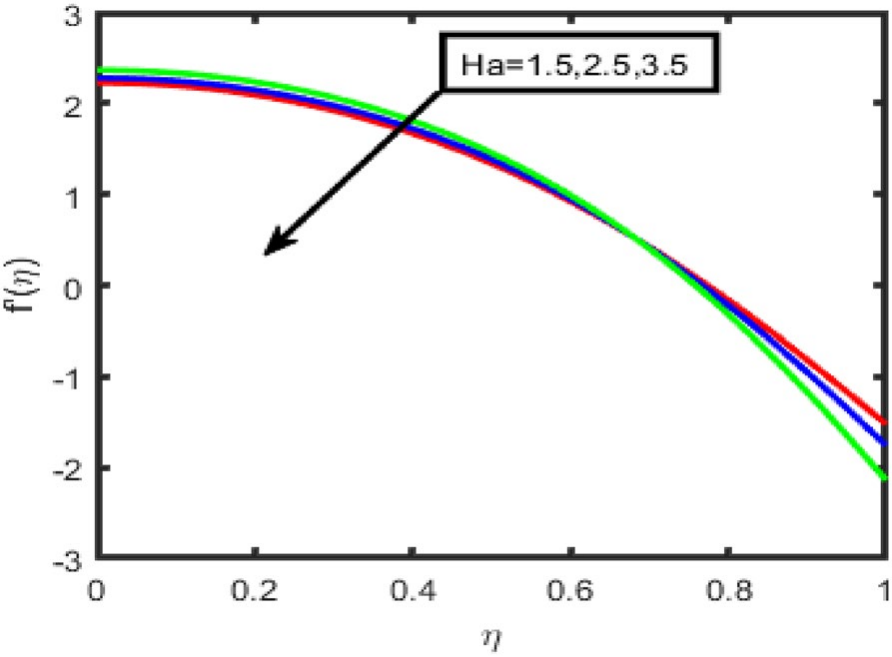
Effect of Ha on velocity.

**Figure 5:**
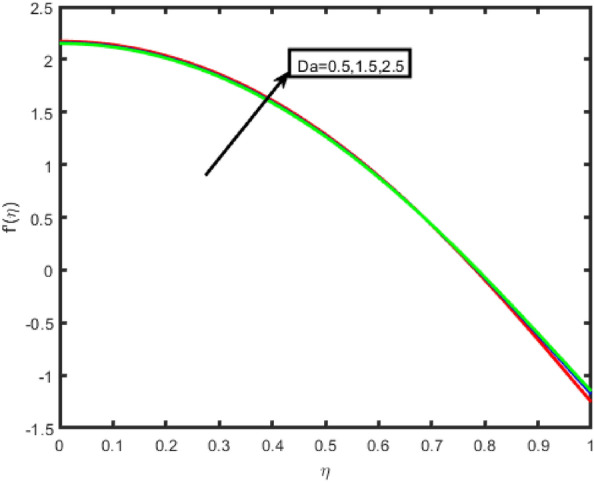
Effect of Da on velocity.

The influence of the Forchheimer parameter (Fr) on the velocity and the temperature profile is seen in Figs. 2 and 3, respectively. It should be noted that the decrease in fluid flow velocity occurs when the value of Fr is increased. Figure 3 demonstrates that an increase in the value of the Frochheimer parameter increases both the thermal gradients and its corresponding boundary layer thickness. The rise in the Forchheimer parameter signifies that the resistive forces are generated within the system. This leads to a decrease in the fluid's motion; consequently, the velocity decreases. Additionally, the temperature of the fluid increases due to the drag force, which inclines the thermal gradients. An increase in the Hartmann number results in a reduction in the standard component of the velocity profile in the flow zone is shown clearly in Fig. 4. It is reasonable to anticipate that a negligible increase in the Hartmann number leads to the increased Lorentz forces associated with magnetic fields. Because of this strong Lorentz forces, a resistance is offered to the fluid passage through the channel. As a consequence, the velocity field will become less intense as the value of the Hartmann number rises. The effect of the porosity parameter on the velocity is seen in Fig. 5. It has been discovered that a more considerable porosity value results in a fluid with a lower velocity and a higher temperature and concentration.

**Figure 6:**
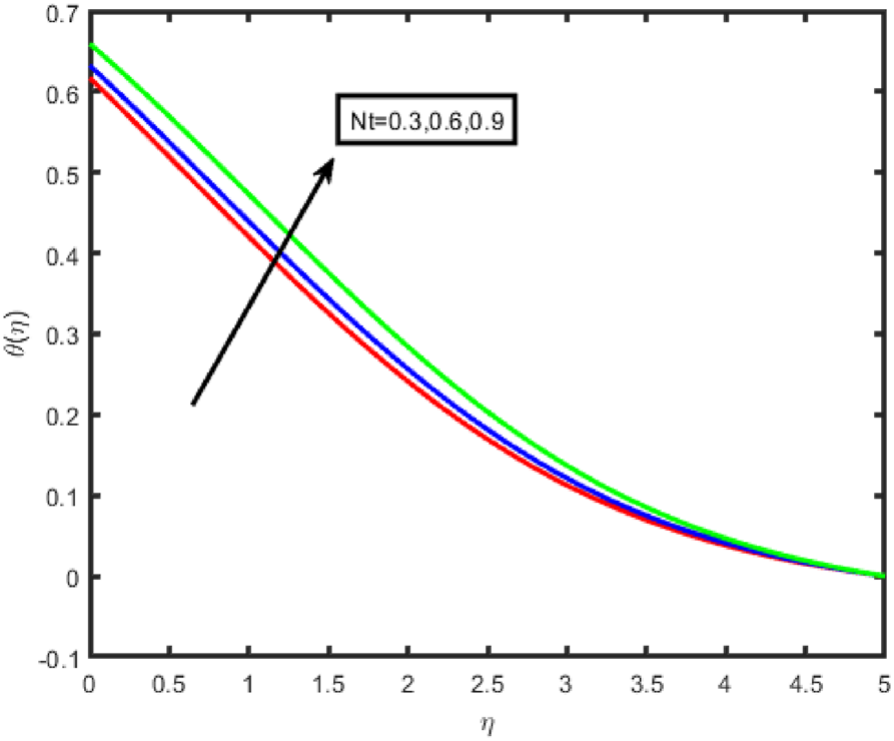
Effect of Nt on temperature.

**Figure 7:**
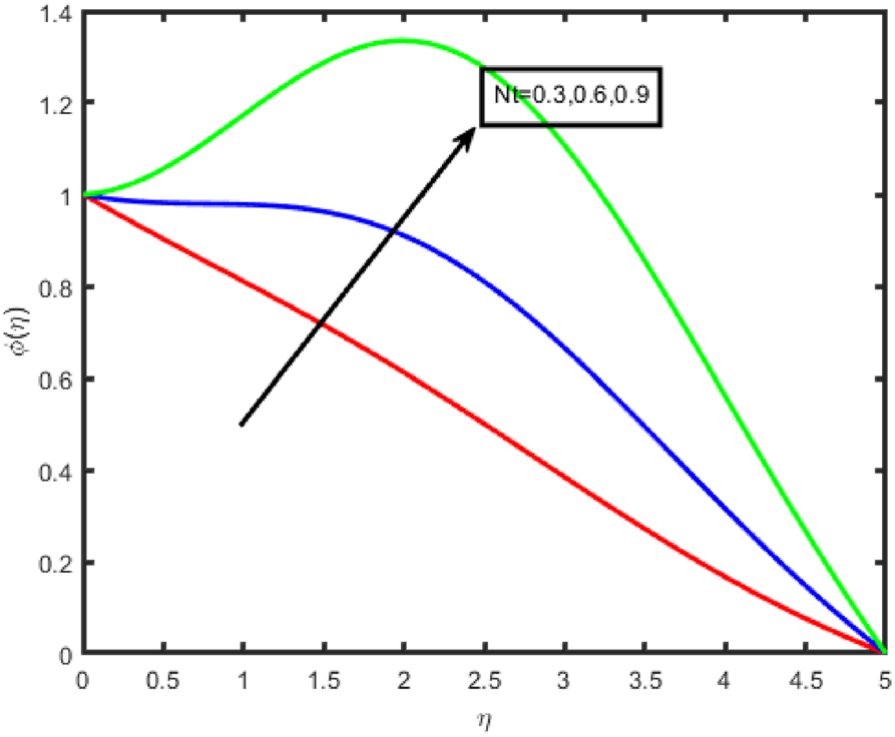
Effect of Nt on concentration.

**Figure 8:**
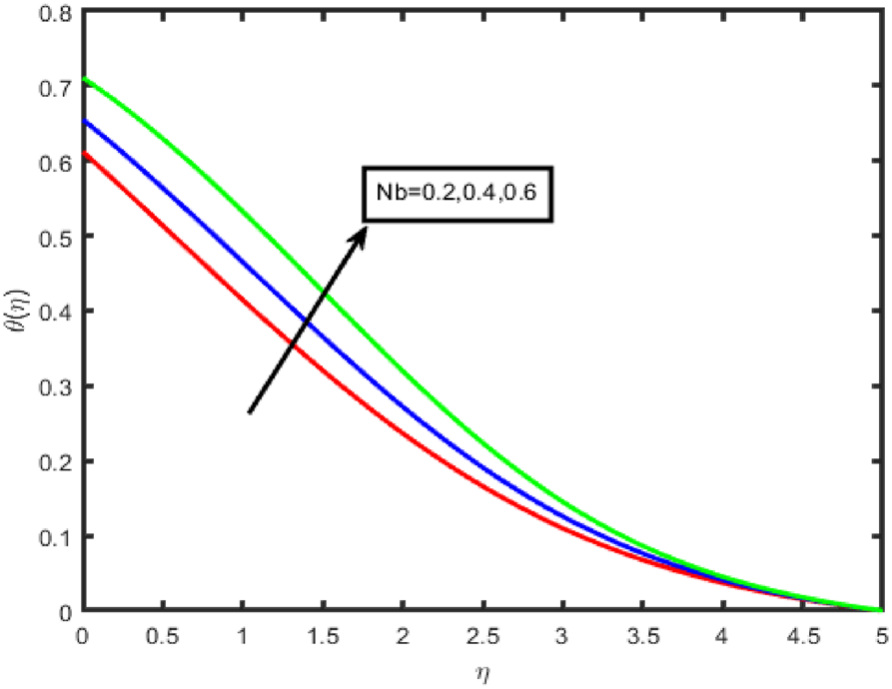
Effect of Nb on temperature.

Figures 6 and 7 illustrate the influence that the thermophoresis parameter (Nt) on the temperature and the concentration, respectively. Temperature and concentration both increase when there is an increase in Nt. Physically, the thermophoresis force is assumed otto be stronger as the value of Nt is raised. This is because the nanoparticles are being pushed away from the hot zone and towards the cold area resulting in an increase in the temperature. A rough draught of Fig. 8 is shown here to explain the change in (ϕ(η)) that results from the Brownian motion parameter (Nb). It was found that the correlation coefficient tends to decrease with increasing Nb values. This phenomenon occurred due to the fluid's nanoparticles moving in an erratic pattern.

**Figure 9:**
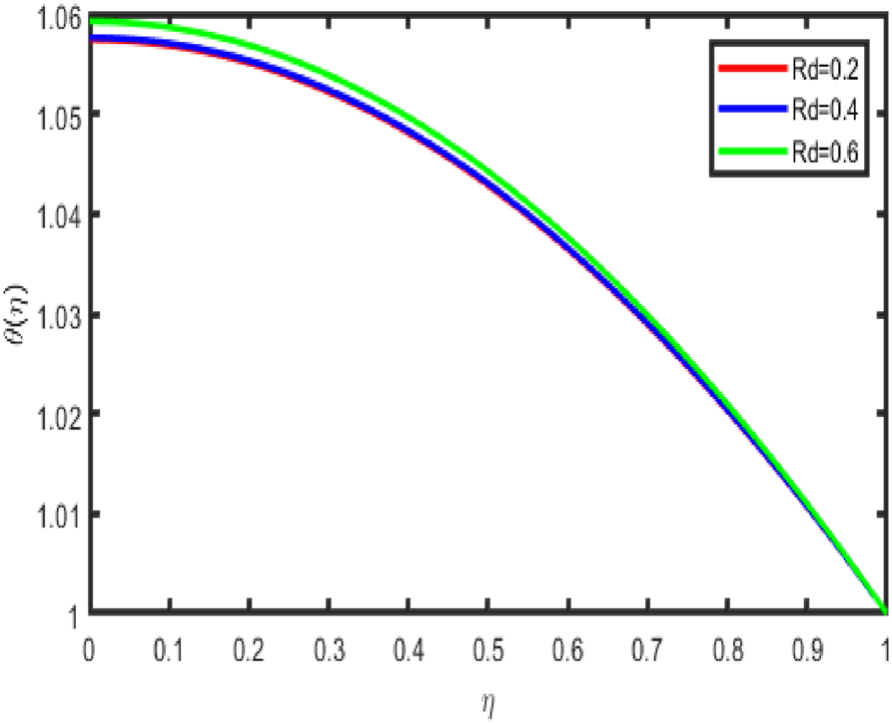
Effect of Rd on temperature.

**Figure 10:**
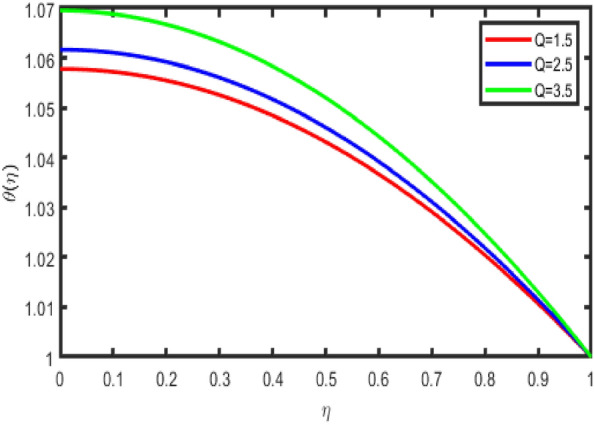
Impact of Q on temperature.

The relationship between an increase in the thermal radiation parameter and a subsequent decrease in the temperature profile in the flow zone is seen quite clearly in Fig. 9. It is possible to draw the following conclusion from Fig. 9. An increase in the thermal radiation parameter results in a higher temperature value, which might benefit various thermodynamic sectors. Figure 10 show how the temperature profile is affected by the effect of the heat production or absorption parameter (Q). It demonstrates an increase in the temperature field when the parameter Q is increased. In addition, the thickness of the thermal boundary layer grows with an increase in the value of Q. It is to be anticipated that, in the process that generates heat, a higher temperature will typically be discharged into the working fluid. As a consequence of this factor, the heat production parameter increases the temperature profile. Additionally, a temperature rise may be attributed to exothermic chemical processes.

**Figure 11:**
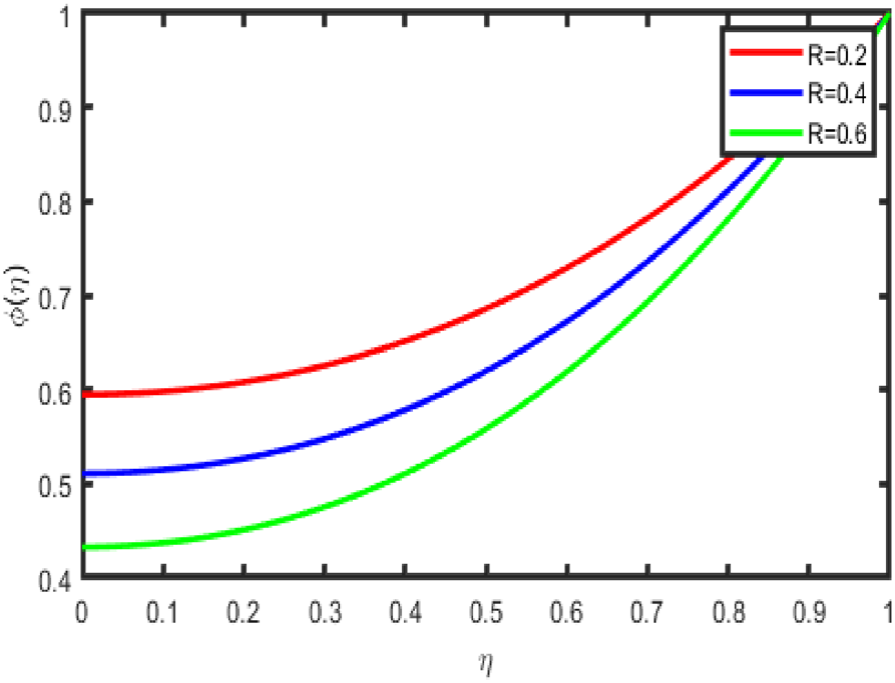
Impact of R on concentration.

**Figure 12:**
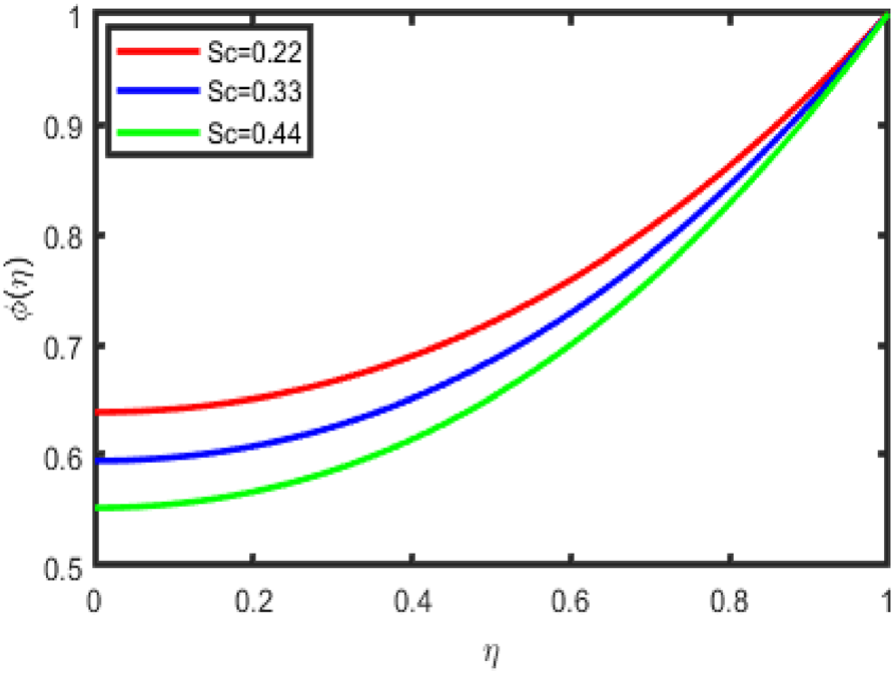
Impact of Sc on concentration.

**Figure 13:**
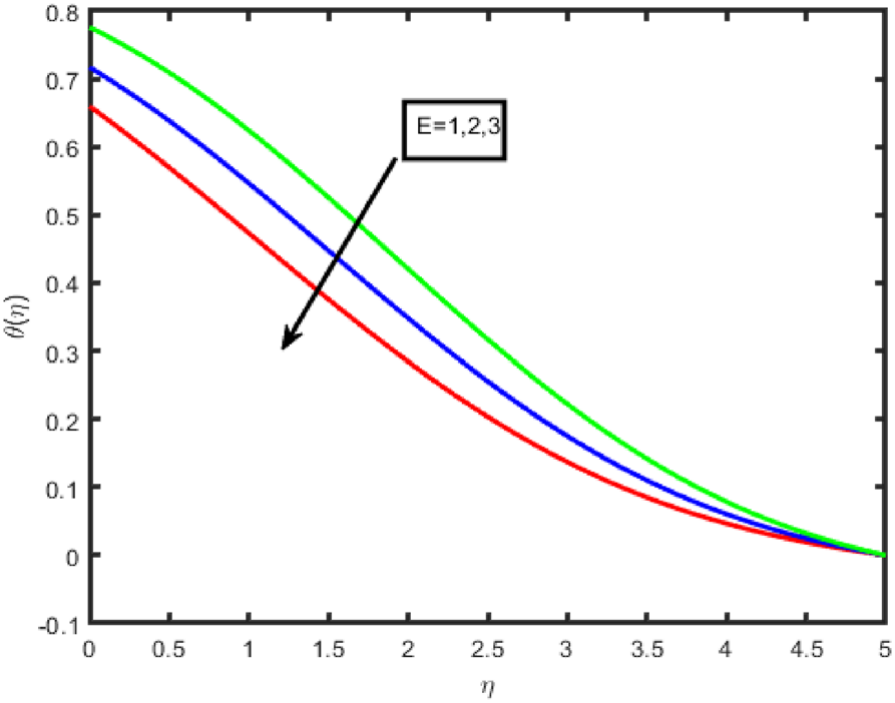
Impact of E on concentration.

Figure 11 is an illustration of the influence that the chemical reaction parameter on the concentration profile. In general, it has been shown that in many instances, a lower concentration field is found for destructive chemical reactions. Figure 12 demonstrates that an increase in the Schmidt number results in a reduction in the concentration field in the flow zone. A slight increase in the Schmidt number reduces the coefficient of mass diffusion, bringing about drops in the concentration field inside the flow zone. In addition to this, it has been shown that the concentration field is a function that decreases as Sc increases. Additionally, when the concentration of the boundary layer increases, the thickness of the coating decreases. Figure 13 discusses the impact that activation energy, denoted by E, has on concentration, represented by (ϕ(η)). The increasing the values of E lowers the Arrhenius energy function, as a result, increasing the rate of the generative chemical reaction that enhances the concentration.

## Conclusions

In the present study, the radiative Darcy–Forchheimer squeezed flow of unstable magneto-hydrodynamic non-Newtonian Casson fluid between two parallel plates with Joule heating and heat production or absorption in the presence of Activation energy and homogenous chemical reaction effects is reported. In the context of the current issue, flow is brought about by the movement of parallel plates. After obtaining the findings of the non-Newtonian Casson fluid flow model, the authors solve the highly nonlinear coupled two-dimensional unsteady partial differential equations by using the classic Runge–Kutta fourth order integration approach in conjunction with the shot technique. Refs.^39–48^ indicates some latest and modern studies on fluid flow, materials analysis and different computational and numerical techniques. The numerical simulations are carried out for each of the many control parameters that have been selected. The following significant inferences may be made based on the previous numerical simulations.

(i) Since the Lorentz forces become more powerful for higher Hartmann number, the velocity decreases.
(ii) An increase in the Forchheimer parameter causes a decrease in the velocity profile and an inclination in the temperature gradients.
(iii) The higher activation energy decreases the concentration profile significantly.
(iv) A rise in the value of the Schmidt number brings about a fall in the concentration profile.
(v) An increase in the porosity parameter causes a reduction in the velocity of the fluid, as well as an increase in the thermal concentration gradients and the thickness of the boundary layer associated with it.
(vi) The concentration field becomes more intense due to the destructive chemical reaction, while the concentration field becomes less intense due to the constructive chemical response.

## Data Availability

All the data are clearly available in the research work.
